# Supplementary material for: Quantifying previous SARS-CoV-2 infection through mixture modelling of antibody levels
Source: Nat Commun. 2021 Oct 26;12:6196. doi: 10.1038/s41467-021-26452-z (PMC8548402; doi:10.1038/s41467-021-26452-z)
Supplement: Supplementary file 1 — Supplementary Information [file 41467_2021_26452_MOESM1_ESM.pdf]

Supplementary Materials for

Quantifying previous SARS-CoV-2 infection through mixture modelling of antibody levels

C. Bottomley, M. Otiende, S. Uyoga, K. Gallagher, E.W. Kagucia, A.O. Etyang, D. Mugo, J. Gitonga, H. Karanja, J. Nyagwange, I.M.O. Adetifa, A. Agweyu, D.J. Nokes, G.M. Warimwe, J.A.G. Scott.

Correspondence to: christian.bottomley@lshtm.ac.uk

This file includes:

Supplementary Tables

Supplementary Figures

Supplementary Notes (Stan code)

Supplementary Table 1: Mixture model parameter estimates and predicted sensitivity and specificity (threshold: OD ratio > 2).

	Uninfected		Infected									
	**Mean**	95% CI	**Mean**	95% CI	**Scale**	95% CI	**Skew**	95% CI	**Sensitivity**	95% CI	**Specificity**	95% CI
**Blood donors**												
Central	-0.17	-0.24 , -0.11	1.76	0.76 , 2.79	1.70	1.00 , 2.67	0.11	-1.79 , 2.13	0.70	0.43 , 0.96	1	1 , 1
Coast	-0.54	-0.60 , -0.48	1.05	0.70 , 1.43	1.34	1.02 , 1.79	0.45	-1.15 , 2.46	0.51	0.38 , 0.66	1	1 , 1
E.N.	-0.16	-0.20 , -0.12	1.16	0.64 , 1.77	1.49	1.09 , 2.01	0.49	-1.35 , 2.59	0.54	0.36 , 0.76	1	1 , 1
Mombasa	-0.72	-0.76 , -0.69	1.95	1.70 , 2.18	1.43	1.04 , 1.93	-0.96	-2.61 , 0.79	0.80	0.72 , 0.87	1	1 , 1
Nairobi	-0.39	-0.46 , -0.33	1.71	1.37 , 2.05	1.47	1.16 , 1.95	0.30	-1.23 , 2.13	0.71	0.60 , 0.81	1	1 , 1
Nyanza	-0.11	-0.15 , -0.06	1.21	0.74 , 1.71	1.50	1.10 , 1.93	1.24	-0.86 , 3.11	0.55	0.37 , 0.74	1	0.99 , 1
Rift Valley	0.07	0.03 , 0.11	2.33	1.68 , 2.97	1.68	1.18 , 2.38	0.27	-1.49 , 2.26	0.82	0.66 , 0.95	0.99	0.98 , 0.99
Western	0.08	-0.01 , 0.17	2.07	0.65 , 3.35	1.56	0.57 , 3.31	0.01	-1.89 , 2.01	0.78	0.42 , 1.00	0.99	0.98 , 0.99
**Antenatal care attendees**												
Kilifi, Oct	-0.61	-0.67 , -0.55	0.55	-0.12 , 1.33	0.82	0.44 , 1.41	0.02	-0.96 , 1.00	0.30	0.07 , 0.70	1	1 , 1
Kilifi, Dec	-0.25	-0.33 , -0.17	1.48	0.76 , 2.24	1.55	1.02 , 2.30	0.36	-1.53 , 2.36	0.64	0.42 , 0.87	1	1 , 1
Nairobi	-0.38	-0.58 , -0.19	1.37	1.09 , 1.64	1.26	0.98 , 1.63	0.72	-0.91 , 2.35	0.63	0.52 , 0.74	1	1 , 1
**Healthcare workers**												
Busia	0.11	0.06 , 0.16	2.22	1.53 , 2.88	1.62	1.12 , 2.35	0.18	-1.65 , 2.19	0.80	0.63 , 0.95	0.98	0.98 , 0.99
Kilifi	-0.09	-0.15 , -0.02	2.05	1.39 , 2.60	1.31	0.85 , 1.99	0.25	-1.54 , 2.21	0.82	0.61 , 0.97	1	0.99 , 1
Nairobi	-0.04	-0.33 , 0.24	1.17	0.86 , 1.49	1.43	1.13 , 1.87	0.50	-1.09 , 2.37	0.55	0.43 , 0.66	0.99	0.96 , 1
**Truck drivers**												
Busia	0.64	0.51 , 0.75	1.57	1.19 , 1.94	1.17	0.89 , 1.42	1.88	-0.11 , 3.42	0.73	0.54 , 0.91	0.8	0.73 , 0.88
Magarini	0.64	0.35 , 0.88	1.35	0.95 , 1.90	1.04	0.74 , 1.46	1.11	-0.93 , 2.85	0.64	0.45 , 0.88	0.79	0.61 , 0.94
Malaba	0.55	0.42 , 0.69	1.28	0.90 , 1.74	1.20	0.88 , 1.53	1.48	-0.78 , 3.41	0.60	0.40 , 0.80	0.85	0.77 , 0.92

Supplementary Table 2: Estimates of the proportion previously infected.

	N	OD ratio > 2	Adjusted*		Mixture model	
**Blood donors**		*%*	*%*	*95% CI*	*%*	*95% CI*
Central	225	6.2	6.1	2.8 - 10.1	9.9	4.9 - 17.2
Coast	435	15.4	15.9	12.2 - 20.0	31.3	23.7 - 40.3
Eastern/ N. Eastern	702	6.0	5.5	3.5 - 7.6	12.2	7.5 – 19.0
Mombasa	802	16.7	17.2	14.3 - 20.3	22.6	19.3 - 26.1
Nairobi	361	23.5	24.8	20.1 - 29.8	35.7	28.9 - 42.9
Nyanza	584	9.4	9.3	6.7 - 12.1	17.6	11.4 - 26.8
Rift Valley	508	8.1	7.8	5.2 - 10.7	8.0	5.4 - 11.3
Western	106	5.7	6.0	1.8 - 11.7	7.3	2.4 - 15.1
All Regions	3,723	11.9	11.9	10.5 - 13.3	44.3	40.4 - 48.3
**Antenatal care attendees**						
Kilifi	264	1.1	0.9	0 - 2.8	5.9	1.7 - 12.9
Kilifi	155	10.3	10.7	5.9 - 16.5	16.3	9.2 - 25.4
Nairobi	196	46.4	49.8	42.0 - 57.8	75.5	63.8 - 87.4
**Healthcare workers**						
Busia	301	12.3	12.6	8.6 - 17.1	12.4	8.0 - 18.0
Kilifi	200	11.5	12.2	7.7 - 17.6	13.5	8.4 - 19.9
Nairobi	183	41.0	43.9	36.0 - 52.0	74.6	59.7 - 92.3
**Truck drivers**						
Busia	365	44.7	48.0	42.1 - 54.0	46.9	29.9 - 74.2
Magarini	101	42.6	45.6	35.1 - 56.3	50.9	20.3 - 91.3
Malaba	364	33.8	35.9	30.6 - 41.8	42.2	23.3 - 71.4

*Adjusted for sensitivity and specificity of the threshold (OD ratio > 2)

Supplementary Table 3: Three tests of the mixture model using data where the proportion previously infected is known.

	Data			Mixture model estimate		
	Uninfected	*Infected*	*% Infected*	*Uninfected*	*Infected*	*% Infected*
**Test 1**: PCR +ve (symptomatic and asymptomatic) and pre-COVID-19 samples combined	N = 910<br>Mean = -0.17<br>SD = 0.42	N = 147<br>Mean = 3.07<br>SD = 1.32	**14%**	Mean = -0.19	Mean = 3.06<br>Scale = 1.72<br>Skew = -2.25	**15%**
**Test 2**: Mixture of distributions in previously infected individuals (simulated data)	N = 200<br>Mean = 0<br>SD = 0.42	N = 25, 25<br>Mean = 2, 0.5<br>SD = 1.4, 1.4	**20%**	Mean = -0.01	Mean = 1.38<br>Scale = 2.06<br>Skew = -0.29	**19%**
**Test 3**: Mixture of distributions in previously uninfected individuals (simulated data)	N = 100, 100<br>Mean = -0.3, 0.3<br>SD = 0.42, 0.42	N = 50<br>Mean = 1.4<br>SD = 1.4	**20%**	Mean = -0.03	Mean = 0.86<br>Scale = 1.67<br>Skew = 0.52	**36%**

Supplementary Figure 1: ROC curves predicted by the mixture model.

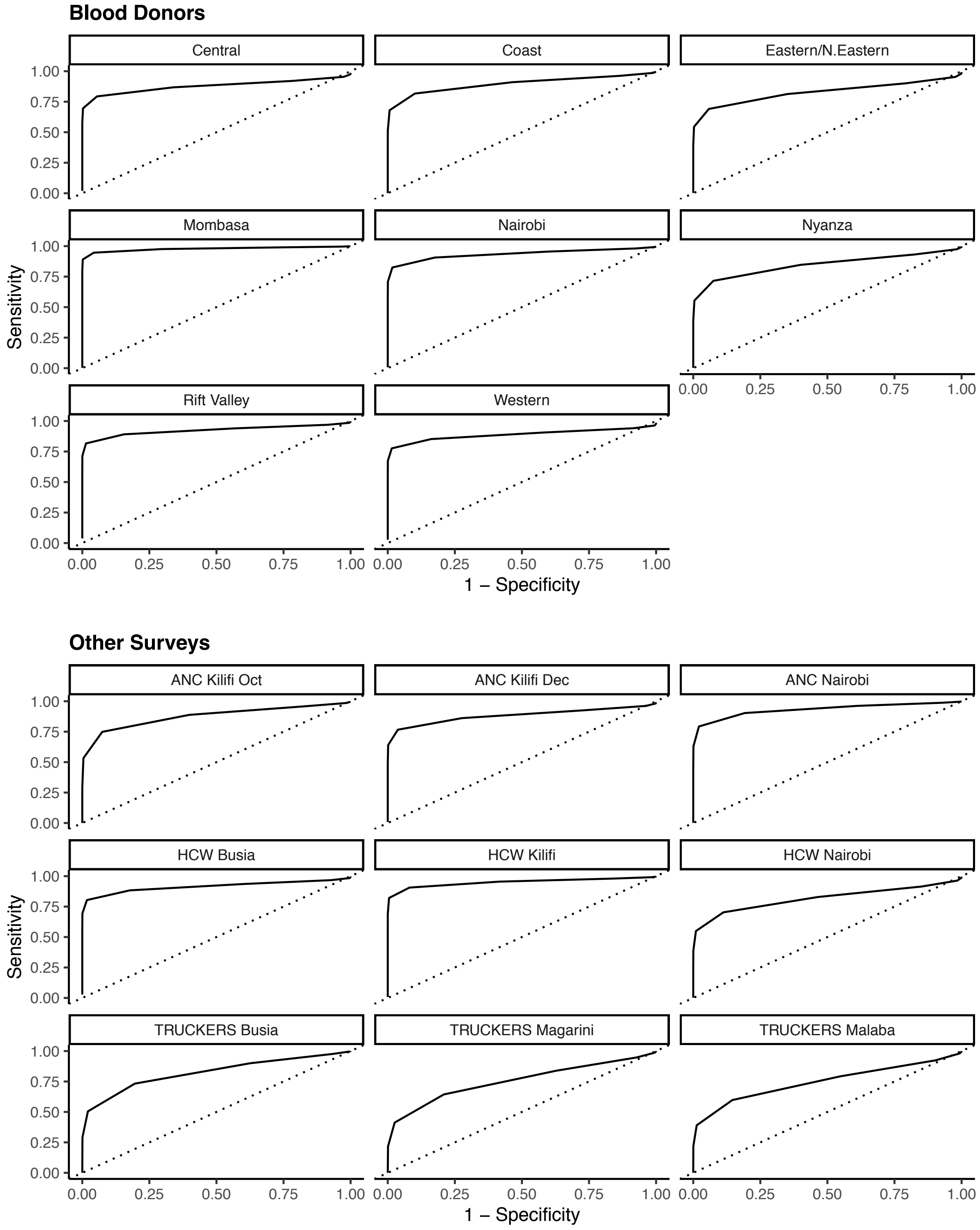

Supplementary Figure 2: Antibody level distributions predicted by the three-component mixture model (blue distribution = previously uninfected individuals, dark/light red distribution = previously infected individuals).

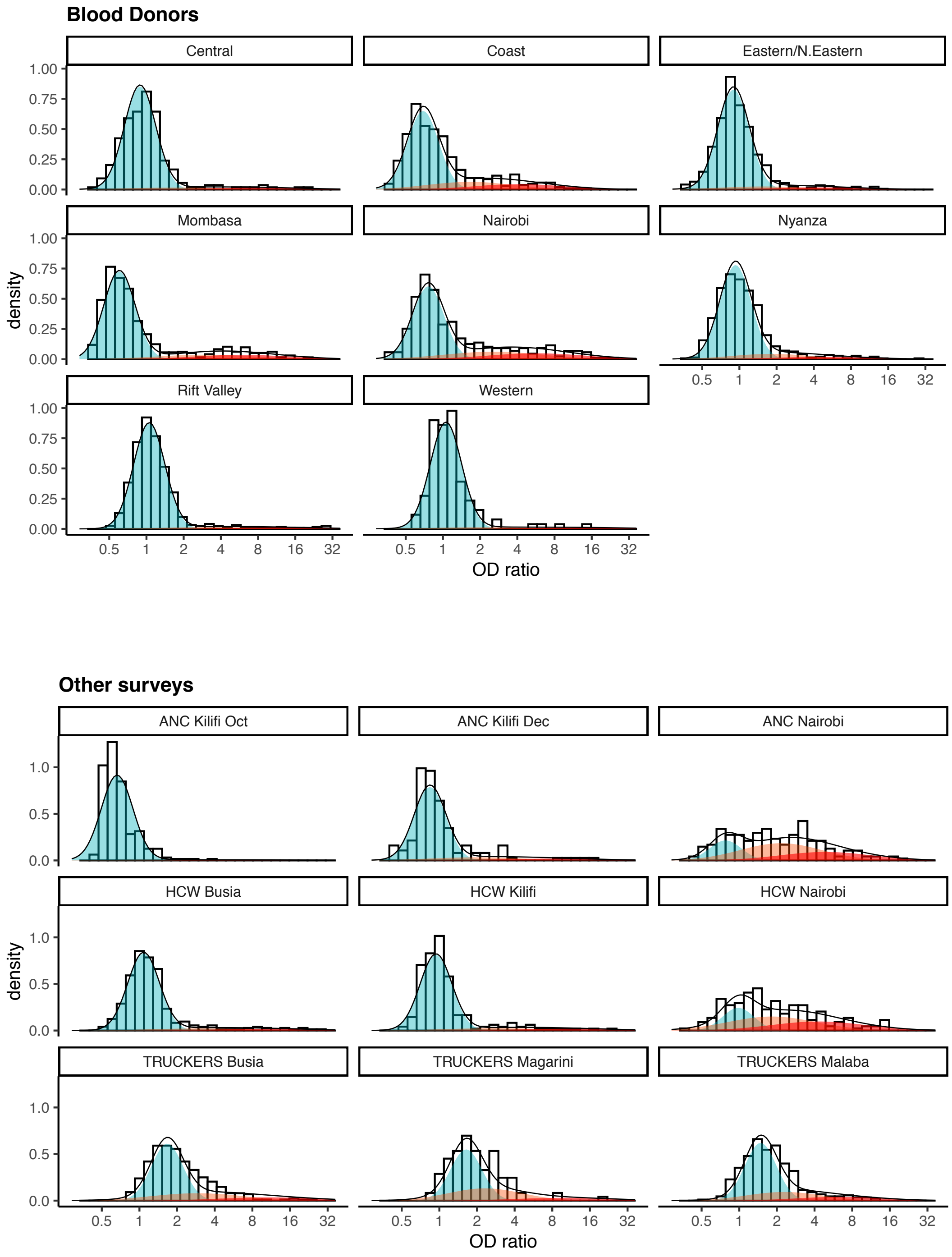

Supplementary Figure 3: Estimates of the proportion previously infected (with 95% credible intervals) from alternative versions of the mixture model.

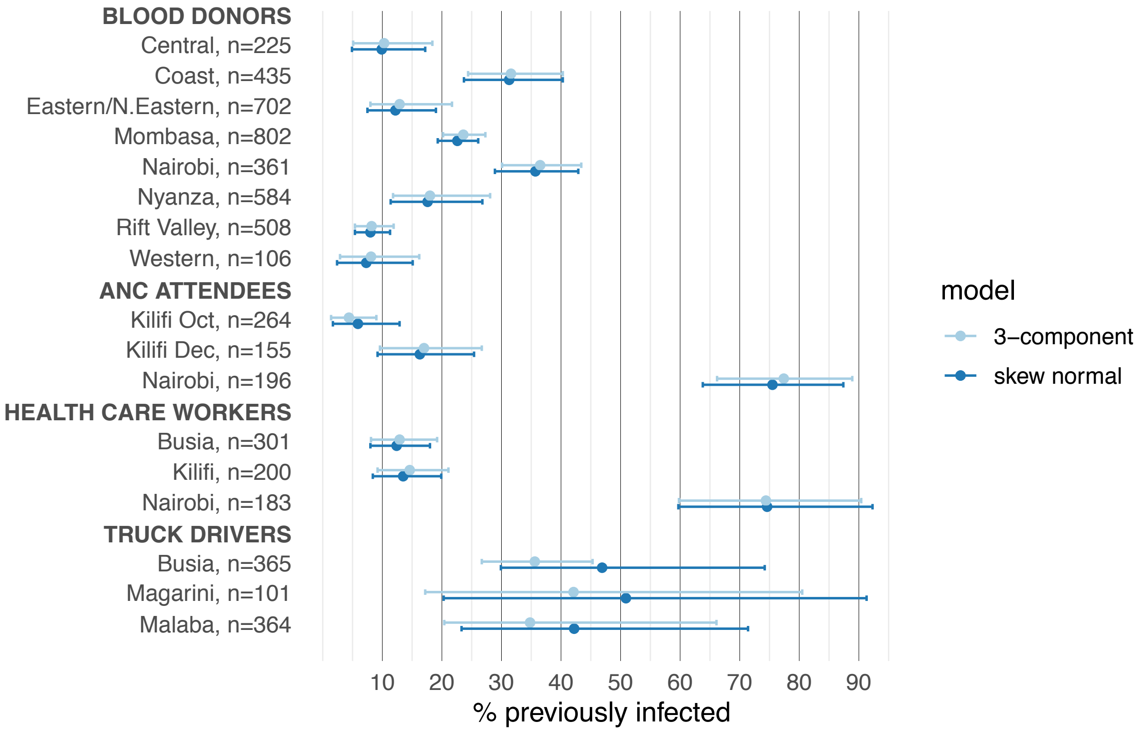

Supplementary Figure 4: Timeline of serosurveys and cumulative COVID-19 cases in Kenya (March – December, 2020).

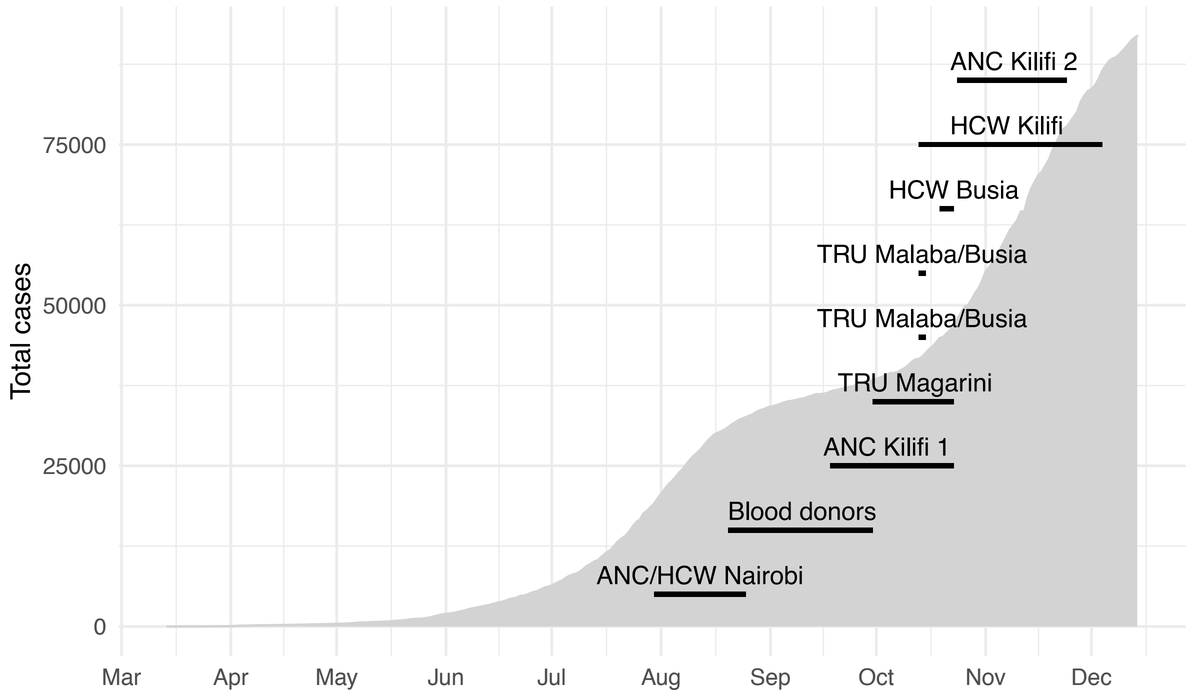

TRU = truck drivers, ANC = antenatal clinic attendees, HCW = healthcare workers

COVID-19 incidence data downloaded from:

https://www.ecdc.europa.eu/en/publications-data/download-todays-data-geographic-distribution-covid-19-cases-worldwide

Supplementary Note 1: Stan code for the threshold analysis adjusted for sensitivity and specificity.

```
data {
  int N;
  int N_se;
  int N_sp;
  int y;
  int x;
  int z;
}

parameters {
  real<lower=0,upper=1> p;
  real<lower=0,upper=1> se;
  real<lower=0,upper=1> sp;
}

transformed parameters {
  real<lower=0,upper=1> p_obs;
  p_obs = se * p + (1 - sp) * (1 - p);
}

model {
   y ~ binomial(N, p_obs);
   x ~ binomial(N_se, se);
   z ~ binomial(N_sp, sp);
}
```

Supplementary Note 2: Stan code for the skew normal mixture model.

```
data {
  int N;
  vector[N] y;
}

parameters {
  real theta;
  real<lower=0> delta;
  real log_omega;
  real alpha;
  real<lower=0,upper=1> p;
}

transformed parameters {
  real xi;
  real nu = 0.42;
  real<lower=0> omega;
  real theta_delta;
  omega = exp(log_omega);
  theta_delta = theta + delta;
  xi = theta + delta - sqrt(2/3.142) * omega * alpha/sqrt(1 + alpha^2);
}

model {

  // priors
  theta ~ normal(0, 10);
  delta ~ normal(0, 1.83);
  log_omega ~ normal(0, 10);
  alpha ~ normal(0, 1);

  //likelihood
  for (n in 1:N) {
    target += log_sum_exp(log(p)
                      + skew_normal_lpdf(y[n] | xi, omega, alpha),
                     log1m(p)
                     + normal_lpdf(y[n] | theta, nu));
  }
}

generated quantities{
    vector[10] cut = [-2, -1.5, -1, -0.5, 0, 0.5, 1, 1.5, 2, 5]';
    vector[10] sp;
    vector[10] se;
    for (i in 1:10) {
      sp[i] = exp(normal_lcdf(cut[i] | theta, nu));
      se[i] = exp(skew_normal_lccdf(cut[i] | xi, omega, alpha));
  }
}
```

Supplementary Note 3: Stan code for the three-component mixture model.

```
data {
  int N;
  vector[N] y;
}

parameters {
  ordered[2] thetaInf;
  real<lower=0> delta;
  vector<lower=0>[2] nuInf;
  real<lower=0, upper=1> p;
  real<lower=0, upper=1> q;
}

transformed parameters{
  vector[3] theta;
  vector[3] nu;
  vector[3] pr;
  theta[1] = q * thetaInf[1] + (1 - q) * thetaInf[2] - delta;
  theta[2] = thetaInf[1];
  theta[3] = thetaInf[2];
  nu[1] = 0.42;
  nu[2] = nuInf[1];
  nu[3] = nuInf[2];
  pr[1] = 1 - p;
  pr[2] = p * q;
  pr[3] = p * (1 - q);
}

model {

  vector[3] log_pr= log(pr);

  // priors
  thetaInf ~ normal(0, 10);
  delta ~ normal(0, 1.83);
  nuInf ~ lognormal(0.28, 0.2);

  //likelihood
  for (n in 1:N) {
      vector[3] lps = log_pr;
      for (k in 1:3){
        lps[k] += normal_lpdf(y[n] | theta[k], nu[k]);
      }
      target += log_sum_exp(lps);
    }
}
```
