# Supplementary material for: Quantifying previous SARS-CoV-2 infection through mixture modelling of antibody levels
Source: Nat Commun. 2021 Oct 26;12:6196. doi: 10.1038/s41467-021-26452-z (PMC8548402; doi:10.1038/s41467-021-26452-z)
Supplement: Supplementary file 3 — Reporting Summary [file 41467_2021_26452_MOESM3_ESM.pdf]

nature portfolio

Corresponding author(s): Christian Bottomley

Last updated by author(s): Sep 13, 2021

Reporting Summary

Nature Portfolio wishes to improve the reproducibility of the work that we publish. This form provides structure for consistency and transparency in reporting. For further information on Nature Portfolio policies, see our Editorial Policies and the Editorial Policy Checklist.

Statistics

For all statistical analyses, confirm that the following items are present in the figure legend, table legend, main text, or Methods section.

n/a	Confirmed	
☐	☒	The exact sample size (*n*) for each experimental group/condition, given as a discrete number and unit of measurement
☒	☐	A statement on whether measurements were taken from distinct samples or whether the same sample was measured repeatedly
☒	☐	The statistical test(s) used AND whether they are one- or two-sided<br>*Only common tests should be described solely by name; describe more complex techniques in the Methods section.*
☒	☐	A description of all covariates tested
☐	☒	A description of any assumptions or corrections, such as tests of normality and adjustment for multiple comparisons
☐	☒	A full description of the statistical parameters including central tendency (e.g. means) or other basic estimates (e.g. regression coefficient) AND variation (e.g. standard deviation) or associated estimates of uncertainty (e.g. confidence intervals)
☒	☐	For null hypothesis testing, the test statistic (e.g. *F*, *t*, *r*) with confidence intervals, effect sizes, degrees of freedom and *P* value noted<br>*Give P values as exact values whenever suitable.*
☐	☒	For Bayesian analysis, information on the choice of priors and Markov chain Monte Carlo settings
☒	☐	For hierarchical and complex designs, identification of the appropriate level for tests and full reporting of outcomes
☒	☐	Estimates of effect sizes (e.g. Cohen's *d*, Pearson's *r*), indicating how they were calculated

Our web collection on statistics for biologists contains articles on many of the points above.

Software and code

Policy information about availability of computer code

Data collection	No software was used in the data collection process.
Data analysis	Data analysis was done in R (version 4.0.4) and the Bayesian models were fitted using Rstan (version 2.21.2). The Stan code is provided in the supplementary materials and R/Stan code for all analyses, including the generation of tables and figures, is available at: https://github.com/christian-bottomley/mixture_model_sarscov2

For manuscripts utilizing custom algorithms or software that are central to the research but not yet described in published literature, software must be made available to editors and reviewers. We strongly encourage code deposition in a community repository (e.g. GitHub). See the Nature Portfolio guidelines for submitting code & software for further information.

Data

Policy information about availability of data

All manuscripts must include a data availability statement. This statement should provide the following information, where applicable:

- Accession codes, unique identifiers, or web links for publicly available datasets
- A description of any restrictions on data availability
- For clinical datasets or third party data, please ensure that the statement adheres to our policy

Source data are provided in the supplementary materials (xlsx file).

Field-specific reporting

Please select the one below that is the best fit for your research. If you are not sure, read the appropriate sections before making your selection.

☒ Life sciences ☐ Behavioural & social sciences ☐ Ecological, evolutionary & environmental sciences

For a reference copy of the document with all sections, see nature.com/documents/nr-reporting-summary-flat.pdf

Life sciences study design

All studies must disclose on these points even when the disclosure is negative.

Sample size	The study was a secondary analysis of data from several serosurveys. The sample size was therefore determined by the size of these surveys. In the original surveys, the sample sizes were chosen to provide reasonable accuracy for the estimation of the proportion seropositive. For example, in the survey of health care workers it was assumed that the seroprevalence at each of the study sites would be 5-10%, and it was estimated that 180 participants per site would generate prevalence estimates with a precision of ± 0.05 to ±0.10 for the site-specific estimates and a precision of ±0.01 to ±0.03 for the overall prevalence.
Data exclusions	In the survey of blood donors samples missing demographic data (2.7%) were excluded. No samples were excluded in the other surveys.
Replication	Assays on a subset of test samples were repeated at least once on separate days and reproducibility confirmed. Positive and negative control samples were routinely included in all runs and the results from these were reproducible.
Randomization	There was no randomization because this was an observational study
Blinding	There was no blinding because this was an observational study

Reporting for specific materials, systems and methods

We require information from authors about some types of materials, experimental systems and methods used in many studies. Here, indicate whether each material, system or method listed is relevant to your study. If you are not sure if a list item applies to your research, read the appropriate section before selecting a response.

Materials & experimental systems

n/a	Involved in the study	
☐	☒	Antibodies
☒	☐	Eukaryotic cell lines
☒	☐	Palaeontology and archaeology
☒	☐	Animals and other organisms
☐	☒	Human research participants
☒	☐	Clinical data
☒	☐	Dual use research of concern

Methods

n/a	Involved in the study	
☒	☐	ChIP-seq
☒	☐	Flow cytometry
☒	☐	MRI-based neuroimaging

Antibodies

Antibodies used	CR3022 monoclonal antibody (mAb), anti-SARS-CoV-2 Ab (NIBSC code 20/130), and the convalescent plasma panel NIBSC code 20/118. These were all used at 1:800 dilution, i.e. same dilution as the test samples. CR3022 mAb was produced in-house using plasmids from Krammer (as described in https://science.sciencemag.org/content/sci/suppl/2020/11/10/science.abe1916.DC1/abe1916-Uyoga-SM.pdf). NIBSC 20/130 and NIBSC 20/118 were from National Institute of Biological Standards and Control (NIBSC) UK.
Validation	The SARS-CoV-2 antibody assay was developed and validated as reported in full in https://science.sciencemag.org/content/sci/suppl/2020/11/10/science.abe1916.DC1/abe1916-Uyoga-SM.pdf.<br><br>The assay was originally validated using 910 pre-COVID-19 serum samples collected in 2018, all of which were collected from adults and children from the Coast region of the country, and samples from 174 PCR-positive Kenyan adults, which were collected from patients admitted to Kenyatta National Hospital in Nairobi and their contacts (14 pre-symptomatic, 55 symptomatic, 92 asymptomatic and 13 unknown).

March 2021

Human research participants

Policy information about studies involving human research participants

Population characteristics	The serological surveys were done in populations of healthy adult Kenyans (blood donors, healthcare workers, truck drivers, antenatal care attendees).
Recruitment	Blood donors:<br>Transfusion samples were collected from four Kenya National Blood Transfusion Service (KNBTS) regional blood transfusion centers that are supported by several satellites and hospitals. The majority of blood donors in Kenya are family replacement donors who provide a unit of blood in compensation for transfusion of a sick relative.<br><br>Healthcare workers:<br>A convenience sample of healthcare workers was recruited at each of the study sites through word of mouth, advertising at hospital notice boards and messages sent via mobile phone. Health care workers of all cadres were eligible to participate in the study.<br><br>Truck Drivers:<br>Truck drivers and their assistants were eligible to participate in the study if they were aged ≥18 years without a contraindication for blood sample collection. In Magarini, drivers transporting salt were engaged whilst waiting for salt to be loaded onto their trucks. In Busia, drivers were engaged at the One Stop Border Posts as they presented for nasopharyngeal and oropharyngeal sample collection for SARS-CoV-2 nucleic acid testing as mandated by the Kenyan government.<br><br>Antenatal care attendees:<br>Samples were collected from all women who provided a routine blood sample at their first visit to the antenatal care clinic.<br><br>We believe the samples are representative of the respective populations. Whether or not the samples are representative of the wider community is less clear. E.g. it is unclear whether the rate of seropositivity in pregnant women in Kilifi reflects seropositivity of adults in Kilifi. However, selection bias is not a significant concern for this particular study because the aim is to compare different methods for estimating cumulative incidence, and such a comparison is possible even if the samples are not representative of the wider population.
Ethics oversight	The protocols of the component studies were approved by the Scientific and Ethics Review Unit (SERU) of the Kenya Medical Research Institute. In addition, approval to publish the results of the antenatal care surveillance was explicitly requested from and granted by Kenyatta National Hospital, University of Nairobi Ethics Review Committee (Protocol P327/06/2020) and the Kilifi County health management rapid response team and SERU.

Note that full information on the approval of the study protocol must also be provided in the manuscript.
